# Madagascar rural observatory surveys, a longitudinal dataset on household living conditions 1995–2015

**DOI:** 10.1038/s41597-024-03879-9

**Published:** 2024-09-30

**Authors:** Velomalala Solo Andrianjafindrainibe, Nicole Andrianirina, Florent Bédécarrats, Isabelle Droy, Jean-Luc Dubois, Jeanne de Montalembert, Bako Nirina Rabevohitra, Rolland Rafidimanana, Patrick Rasolofo, Raphaël Ratovoarinony, Lalasoa Anjarafara Onivola Ratsaramiarina, Jean Dieudonné Ravelonandro, Voahirana Razanamavo, Mireille Razafindrakoto, Bezaka Rivolala, François Roubaud, Camille Saint-Macary

**Affiliations:** 1 Independent Researcher, Antananarivo, Madagascar; 2 French National Research Institute for Sustainable Development (IRD), Paris, France; 3 UMI SOURCE (Paris-Saclay University, UVSQ, IRD), Paris, France; 4 UMR LEDa-DIAL (IRD, PSL University, Paris-Dauphine University, CNRS), Paris, France; 5 Unit for emergency prevention and management (CPGU), Prime Minister Office, Antananarivo, Madagascar; 6 International Institute for Social Sciences (IISS), Antananarivo, Madagascar; 7 Malagasy Institute for Planification Techniques (IMATeP), Antananarivo, Madagascar

**Keywords:** Social sciences, Developing world, Agriculture, Economics

## Abstract

A Rural Observatory System (ROS) was established in Madagascar to address the lack of socioeconomic data on rural areas. It collected, analyzed, and disseminated data to help formulate and evaluate development policies. From 1995 to 2015, the ROS surveyed a total of 26 areas. The ROS methodology involved annual household panel surveys using consistent questionnaires supplemented by modules covering new themes. Qualitative community surveys were used to understand local features and dynamics. The site selection combined quantitative and qualitative insights to reflect the diversity of Madagascar’s rural challenges. Quality control was comprehensive, with measures such as limiting the number of daily surveyor interviews and daily field supervision. By making this data available for 21 consecutive years, along with documentation, metadata, and code with analysis examples, we aim to facilitate their discovery, assessment, and understanding by researchers, policymakers, and social organizations. To our knowledge, this is the only available data for an in-depth analysis of the situation and trends in the rural areas of Madagascar.

## Background & Summary

Rural households constitute the majority of the population of the least developed countries (64% in 2022) and account for the bulk of worldwide poverty^1^. Recent reductions in poverty rates in developing countries stem from a decrease in rural poverty^2^. Although improving living conditions in the countryside is one of the keys to enhancing welfare in developing countries, rural areas are generally neglected or poorly grasped by national statistical systems^3–7^. In Madagascar, the population has grown from 12 million in 1993 to 26 million in 2018, with 81% of the population living in rural areas^8^. In the mid-1990s, Madagascar’s rural areas underwent profound transformations. After a socialist period from 1975 to 1985, the country moved towards economic liberalization following the IMF recommendations: liberalization of pricing and marketing structures, and a shift from state-driven policies to market-oriented reforms^9^. The existing statistical system, which had deteriorated during the socialist era, was ill-equipped to capture these changes, leaving a gap in understanding the rural landscape evolution^10^. This situation was particularly concerning given that agriculture employed almost 80% of the active population, and that any analysis of the Malagasy economy would be incomplete without a comprehensive understanding of its rural sector.

This lack of information motivated the creation of four rural observatories in Madagascar in 1995^11,12^, each representing distinct agricultural challenges within the country’s diverse ecosystems. The observatories consisted of annual panel survey, dedicated to the continuous collection, analysis and dissemination of socioeconomic data and insights, with the aim of identifying characteristics and dynamics across diverse agroecological contexts to guide the formulation, implementation, and evaluation of rural development policies^13^.

As the ROS emerged as an innovative and cost-effective way to provide a nuanced and continuous observation of rural households, its extension was supported by new funders and partners, such as research institutions, non-governmental organizations or consultancy firms^14^. The institutional anchoring of the rural observatories changed in 2000, moving from the National Statistical Office, with which it was initially associated, to the Rural Development Policy Unit, within the Ministry of Agriculture. A charter was drawn up to promote the methodological unity of the observatories and the ethics of intervention. Each partner involved was required to sign it to ensure methodological consistency across observatories and over the years.

**Fig. 1:**
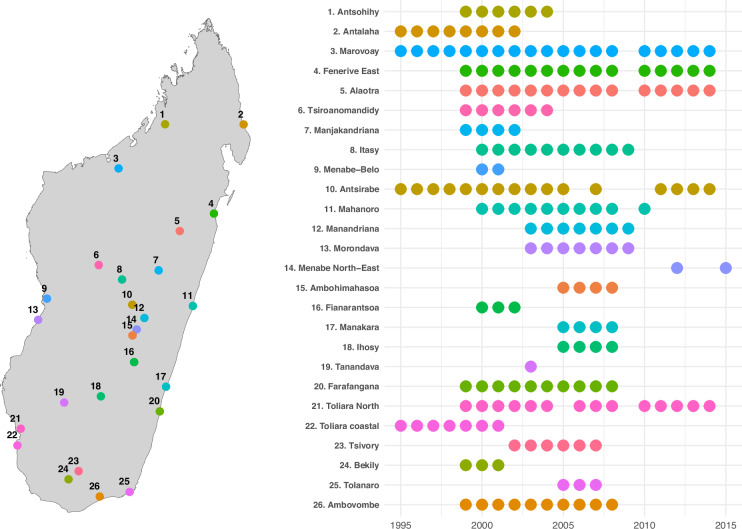
Coarse location and years of data collection of observatories (Source: authors). The figure is composed of two panels. The left panel displays a map of Madagascar with colored and numbered dots corresponding to the locations of the 26 observatories. The right panel indicates, for each number, the name of the observatory and the survey years.

Over the years, the number of observatories increased, reaching 17 in 2004, before contracting to five following the political crisis of 2009^15^. Despite these challenges, the ROS persisted, with three of the original observatories remaining to operate actively until 2014, as shown in Fig. 1. In 2015, a previously surveyed observatory was surveyed again (Menabe North-East, #14 on Fig. 1), and three new observatories were initiated (Ambatofinandrahana, Anjozorobe, and Maintirano, not shown in Fig. 1). Substantial work is required to clean, harmonize, and document the data from these latest observatories to include them in the dataset. The data from these new observatories will be added to the dataset at a later stage after the publication of this data descriptor. The ROS remained operational until 2017

The closure and relocation of observatories primarily resulted from changing priorities of funding organizations and policymakers^16^. For example, the Toliara coastal and Antalaha observatories (#22 and #2) were discontinued when the French Cooperation shifted its focus to new sites established in 1999 and 2000. Toliara Coastal closed because another monitoring system in the area focused on fishing and shrimp production. Similarly, the Antsohihy and Tsiroanomandidy observatories (#1 and #6), funded by the European Union, were halted in 2005 due to a strategic shift towards food security, leading to the new observatories in the southern regions. The Bekily observatory (#24) was closed after a German cooperation food security project ended. The Antsirabe site (#10) suspended operations due to the gradual withdrawal of Norwegian cooperation but was reactivated due to the Agricultural Ministry’s interest in its agro-pastoral system. Rice production has remained a major strategic priority for Madagascar, so observatories focusing on rice production areas, particularly Alaotra (#5) and Marovoay (#3), were continuously sustained. Internal management issues and difficulties in maintaining competent personnel led to the intermittent cessation of operations, as seen with the Tsivory observatory (#23).

The ROS adopted a methodology designed to combine consistency and continuous innovation. Annual surveys were conducted using questionnaires with stable parts that remained largely unchanged, ensuring data continuity on key variables over time. This approach allowed to follow-up households, capturing changes in their socioeconomic conditions and their responses to external shocks and policy changes. Over time, new topics were introduced, and existing questions were enriched to adapt to contextual changes and emerging challenges. These additions focused on key areas such as food availability and feeding practices, particularly during lean periods, exposure to natural disasters, damages, and strategies to recover after shocks, and access to basic services such as potable water, sanitation facilities, education, or health care. However, the survey faced the challenge of ‘questionnaire inflation,’ as the eagerness to add new variables without removing outdated ones significantly extended the average interview duration over the years.

The ROS methodology combined quantitative methods to describe situations and a qualitative perspective to explain them, offering a comprehensive view of rural dynamics. In addition to the household questionnaire, a community survey was conducted, involving field observations and semi-structured interviews. This mixed-method approach produces general information about the region and provides structural and situational insights on various aspects of community life (agricultural campaigns, social and cultural environment, health, education, security, development support, and product pricing).

**Fig. 2:**
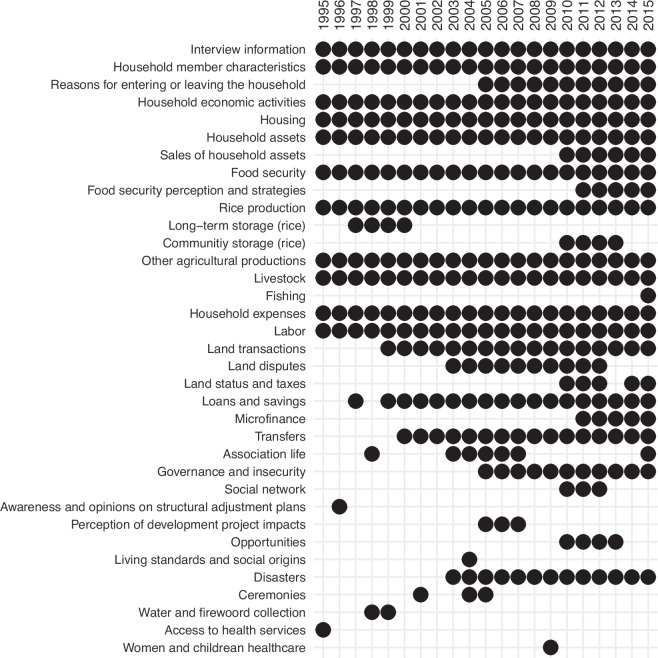
Summary of the main modules included in the household ROS questionnaires each year (Source: authors). The chart illustrates the presence of survey modules across years from 1995 to 2015: each black dot represents the inclusion of a specific module in the survey for that year, while the absence of a dot indicates that the module was not included.

Over the years, the core questionnaire has been updated by adding new modules and removing of others, as shown in Fig. 2. For instance, a module on governance has been included since 2005 to assess the people’s experiences and perceptions of the effectiveness of public services (administration, hospitals, schools, etc.) and insecurity. Another example is the inclusion of a module on maternal and child health in 2009. This module was commissioned by donors in the context of a major political crisis that crippled the national health information system. It was not maintained in the subsequent years. Compared to specific health surveys, such as the Demographic Health Survey, the module included only basic questions on the prevalence of diarrhea, cough and fever, and access to health care. Another module on food security administered throughout the entire period also includes relevant information on health determinants, such as food quantity, quality and diversity. With regard to demographic events, live births have been recorded from the outset within the household member roster. However, data on deaths and outmigration were only collected from 2005 onwards, among the section registering the reasons for members to leave the household.

Retrospective assessments of the cost of the ROS in the 1990s and 2000s have been reported^10,16^. We consolidated this information and supplemented it with more recent accounts. To ensure comparability with 2023 values, historical prices have been adjusted for inflation and the average exchange rate between the respective survey years and 2023. As a result, we find that the typical costs for data collection at the 2023 value range from €25 to €50 per surveyed household. These costs vary according to several factors, such as the remoteness and accessibility of the sites, the availability of public transport, catering and accommodation, and whether the observatory is a new one. These figures do not include costs associated with upstream activities, such as methodological design, or downstream tasks, such as data cleaning, analysis, and dissemination.

Over the years, the ROS data have played an important role in various academic studies, policy formulations, and development projects. The dataset includes a bibliographic database of 120 references, which provides a broad overview of the work that has been derived from this data. The ROS has primarily served as a national instrument for monitoring changes in the countryside and in the living conditions of rural households, enhancing the rural information systems. Researchers from various disciplines, including sociology, economics and agronomy, have used ROS data with both qualitative and quantitative methodologies. These studies cover a range of topics such as poverty dynamics and vulnerability, family structures and food security, and the impact of climate shocks and agricultural policies. Additionally, studies on agricultural value chains and markets provide insights into the challenges and opportunities facing Malagasy farmers. In general, the analyses show that rural households in Madagascar are very poor, have little room for maneuver, and are highly vulnerable to economic or climatic shocks.

The longevity and consistent methodology make it unique among households panel survey experiences. Nevertheless, akin to all survey designs, it has encountered challenges, particularly concerning measurement and sampling, addressed through methodological improvement over time.

## Methods

In a country like Madagascar, where the statistical information system is flawed, the ROS surveys had several advantages that explain why they lasted for several years before successive periods of crisis interrupted the process. They were based on a lightweight, modular organization, using a consistent methodology to generate economies of scale. Rigorous quality control was implemented at every stage, competent supervisors were trained, and institutional capacities were strengthened. Moreover, the ROS was integrated into the national statistical information system, and the results were rapidly published and widely disseminated in the media.

### Site selection

The selection of observatory locations was based on a qualitative approach leveraging the expertise of individuals with extensive knowledge of the field to reflect the diverse array of rural situations. Due to the multiplicity of terrains and climates, Madagascar has diverse ecosystems for agriculture. Farming practices are shaped by the ethnocultural distribution of the population, underscoring the need for rural development policies to consider this diversity. A first subset of potential locations was prioritized to characterize a wide range of agroclimatic areas, production systems, population density, accessibility, and support structures. These potential locations were then correlated with development indicators reflecting their economic and social conditions. The choice was also guided by the interests of donors operating in a given region. The observatory locations were selected from each category to constitute a varied portfolio. Each of the 26 observatories consisted of at least two sites, whether hamlet or village, strategically selected to capture the local diversity. This approach aimed to provide an in-depth understanding of the many facets of the rural landscape.

Table 1 and Table 2 present the diverse range of agricultural and climatic zones encompassed by the ROS, reflecting the country’s rich ecological variety. The observatories cover areas ranging from arid and semi-arid regions with predominant activities in dry crops (maize, tubers) and pastoral farming with some irrigated rice perimeters to humid tropical zones where rice cultivation, fishing, and a mix of subsistence and cash crops like vanilla, coffee, and cloves thrive. This network captures the dynamics of different agricultural practices and climatic challenges and addresses unique regional issues such as food insecurity, land conflicts, and exposure to cyclones.

**Table 1:** Description of Observatories with Names Starting with Letters A to L (Source: authors).

Name and number on Figure 1	Outstanding features
Alaotra #5	Intermediate plateau, one of Madagascar’s main rice baskets.
Ambohimahasoa #15	Tropical highland climate, relatively warm and humid, irrigated rice cultivation, fish farming, some livestock.
Ambovombe #26	Semi-arid region, frequent droughts, recurrent food insecurity, agro-pastoral system in which zebu plays a central role.
Antalaha #2	Family farming with production of rice for family consumption and cash crops (vanilla, coffee, pepper, and cloves).
Antsirabe #10	Small family farming on highlands, tropical highland climate, high.
Antsohihy #1	Diversified agriculture in a semi-humid tropical environment, large rice-growing plains but very isolated region.
Bekily #24	Significant local conflicts and diverse agriculture (rice, maize, peanuts, potatoes, vegetable gardening).
Farafangana #20	Tropical humid climate. Subsistence farming, cash crops (coffee, pepper, cloves), high cyclonic risk.
Fenerive East #4	Humid tropical zone, rice cultivation and cash crops (cloves, vanilla, etc.), high cyclonic risk and fluctuations of cash crops prices on the global market.
Fianarantsoa #16	High-altitude tropical climate, rice cultivation, off-season crops, small livestock farming.
Ihosy #18	Tropical highland climate. Some industrial crops (sugar and tobacco), livestock. Affected by fires and deforestation.
Itasy #8	Central plateau, rich volcanic soils, diversified crops and large plains for rice cultivation, land tenure issues.

**Table 2:** Description of Observatories with Names Starting with Letters M to Z (Source: authors).

Name and number on Fig. 1	Outstanding features
Mahanoro #11	Fishing, cash crops, tourism, high cyclonic risk.
Manakara #17	Hot and humid tropical climate, cyclones, particularly diverse agricultural production, fishing activities.
Manandriana #12	Tropical highland climate. Low-yield rice areas, complementary subsistence crops.
Manjakandriana #7	Diverse agriculture (rice, potatoes, cassava, taro, sweet potatoes, beans, peas) and cattle farming. Tropical highland climate.
Marovoay #3	Large irrigated rice farming area in plains, dry tropical climate.
Menabe-Belo #9	Semi-arid tropical climate with short rainy season, pastoral system and irrigated agriculture, fishing, water scarcity.
Menabe North-East #14	Tropical climate with a dry season, insecurity (cattle theft), rice cultivation, legume production for export, fish farming.
Morondava #13	Semi-arid tropical climate, affected by bushfires, deforestation, erosion, agriculture (mainly irrigated rice), livestock farming.
Tanandava #19	Former area of administered production of cotton and rice. Hot climate with short rainy season.
Tolanaro #25	Increasing maize cultivation competing with rice, significant coffee production, intermediate climatic zone.
Toliara coastal #22	Fishermen and agro-pastoralists in an arid and isolated region.
Toliara North #21	Relatively developed agriculture (rice, cassava, maize, sugarcane) despite a dry climate outside the rainy season.
Tsiroanomandidy #6	Area of recent migration, young population, land issues, persistent insecurity due to armed cattle thieves.
Tsivory #23	Fertile but isolated region, periodic droughts, irrigated rice cultivation, vegetable crops.

We illustrate this heterogeneity by describing the first four observatories established in 1995. The Toliara observatory (#22 on Fig. 1) is located on the coastal Mahafaly Plateau in Toliara Province, an arid and isolated area with a low population density. This observatory was of particular interest due to its landlockedness problem, which is representative of a large part of rural Madagascar (territory and population). However, it is also part of a regional trading system. In addition, the coexistence of two ethnic groups, the Vezo, who rely on fishing, and the Tanalana, who are livestock farmers, is another advantage, as these two populations have different but complementary production systems. The two villages selected, Beheloka and Itampolo, also struggled with primitive living conditions, lack of fresh water, and reduced public services. The area was prone to low and irregular rainfall, leading to frequent droughts and crop failures.

Antalaha observatory (#2), located on the Northeast coast, was characterized by large-scale production of traditional export commodities: vanilla, coffee, pepper, and cloves. The observatory monitored producers’ responses to the liberalization of the vanilla trade. The three villages selected, Maromandia, Tampolo, and Ambohitralalana, represent different levels of landlockedness.

The Vakinankaratra observatory (#10) in Antsirabe, located in the Madagascar central plateau, highlighted the challenges faced by family smallholdings that primarily engage in rice cultivation but experience a rice shortage. The central plateau is the most densely populated region in Madagascar, and the peasant farmers have developed a diversified cropping system to maximize the potential of the varied landscape. This observatory is characterized by a production system in which irrigated rice cultivation plays a central role. Polyculture and polyactivity are other significant features (dairy farming, fruit crops, handicrafts, charcoal-making). In addition, seasonal migratory flows, permanent settlements on the “margins of the highlands”, and less densely populated areas contribute to the region’s complexity. Two villages were selected to reflect the regional diversity: Ambatomena, an old settlement zone, and Vinany, a settlement established by migrants from other regions.

The Marovoay observatory (#3), in the Lower Betsiboka plain, was a significant rice-growing area and belonged to one of the large irrigated perimeters constructed at the beginning of the 20^th^ century^17^. This region was mainly populated by migrants from several regions in search of salaried employment and land. A wide variety of communities could be found on the Marovoay plain. The population is young, and the main activity is farming, with almost all households having a secondary activity, often linked to farming or fishing. Farmers are highly integrated into the market economy. This region was known for its productive rice fields, and even some rice varieties were exported to Europe until the 1970s. It experienced challenges during the socialist period and the 1980s crisis, leading local farmers to adapt through retrenchment or diversification strategies. Two villages were selected in 1995: Ampijoroa and Maroala.

As shown in Fig. 1, the focus in Toliara shifted to a different location at the beginning of the 2000s (#22 to #21), and the Antalaha observatory (#2) was discarded in 2004. The initial limitation to four observatories was due to resource constraints, the experimental nature of the project. The number of observatories expanded during the 2000s, influenced by the operational interests of the ROS donors, while seeking to maintain a similar level of diversity to that illustrated above.

### A panel household survey

For each observatory, an annual, stratified, two-stage survey of households was carried out. The survey adopted the format of a panel study, with the sample of a consistent group of households from year to year. In the initial annual survey conducted in the year of each observatory’s creation, at least 500 households situated in a minimum of two sites were selected. In small localities, the sample may have encompassed all households in the locality, while in more populated places, the sample households were randomly drawn from a comprehensive enumeration of all households in the locality. The sample frame was compiled at the beginning of each annual survey to identify the households that had participated in the previous year and, if applicable, ascertain the reasons behind the attrition of households (death, moving, refusal to answer). By doing so, the survey was able to account for changes in population from one year to the next, such as households relocating and new households being established. To maintain a consistent sample size of 500 households for each observatory and ensure sample representativeness, households that had relocated were substituted by new households through random selection. These new households were sourced from villages already covered by the survey if their overall population was initially not fully included, or from neighboring villages otherwise.

The unit of observation for the rural observatories is the household, not the farm, as in traditional agricultural surveys. This approach allowed for the comprehensive coverage of all activities undertaken by each individual and to establish a crucial, yet often blurred, distinction between the rural sphere and the realm of agriculture. While most rural households are engaged in activities such as crop farming, livestock husbandry, or fishing, others pursue occupations such as craftsmanship, trade, shopkeeping, or wage labor. Some households combine agricultural and non-agricultural activities. In addition, within each household, a distinction was made between the primary activity, which contributes most to income and consumes most of working time, and the secondary activities. It is common for households to participate in several income-generating activities, and their survival often hinges upon this diversification. Similarly, this approach allows for the inclusion of transfer income from family members engaged in long-term migration (over six months), but whose contributions enable the unit to continue its farming activity. Therefore, focusing on households enables the assessment of secondary activity, transfer revenues and identifies the working population holding jobs not necessarily in the agricultural sector.

Data collection relied on statements provided by the household heads and their partners, who responded to qualitative and quantitative questions. Under this system, the extent of cultivated land, the volumes of agricultural production, and the quantities sold were determined based on the knowledge and recollection of the producers. The survey was based on a comprehensive questionnaire designed to capture the multifaceted nature of the rural environment, encompassing diverse crops, livestock, and both agricultural and non-agricultural activities. The questionnaire included a consistent general section, used each year by all observatories annually, but also special sections tailored to specific observatories or themes, like vanilla cultivation or fishing, or highlighted topics like education, gender roles in household tasks, migration, etc. The general section mainly focused on socioeconomic data, including household living standards, children’s education, and food security, as well as income sources, prices, quantities marketed or consumed, and agricultural production factors, offering a holistic view of rural livelihood states and strategies.

The ROS methodology differs in two main ways from other population observatories widespread in Africa, such as the Demographic and Health Surveillance System (HDSS). Firstly, the ROS aims to collect information on socioeconomic factors and agricultural activities while the HDSS focuses primarily on population demographics and health events. Secondly, the sampling strategy is significantly different: the ROS randomly select a sample of 500 households per observatory, whereas the HDSS collects information from the entire census of a geographically defined population^18^. Nonetheless, both ROS and HDSS face a similar limitation due to the longitudinal nature of their data: the ethical challenge of repeatedly tracking households in the sample over a long period.

### Team organization

Conducting surveys across dispersed locations in Madagascar, especially in isolated areas, posed significant logistical challenges. Each observatory’s collection team consisted of two to three supervisors, ten to twelve surveyors, and a driver. The primary supervisor for each observatory, with a background in statistics and survey experience, was supported by one or two deputy supervisors knowledgeable in economics, agricultural science, or geography. Their continuous presence in the villages during survey periods was crucial for effective and reliable data collection.

To foster effective communication, some supervisors and surveyors were locally recruited at each observatory site, ensuring their familiarity with local customs, regional languages, and practices. However, surveyors were never assigned to their native villages to maintain confidentiality. Given the temporary nature of their roles and the unavailability of the same team members each year, annual recruitment and training sessions were essential. These sessions, conducted with local partner organizations, included selection processes and intensive training focusing on the questionnaire objectives, teamwork, and household engagement. Supervisors were trained first, followed by surveyors who were trained by supervisors. Each training session ended with a field test survey.

Before beginning the actual survey, surveyors underwent an “integration stage” for a few days in the assigned villages. During this phase, they liaised with local administrative and traditional authorities, often by presenting previous survey results. The team would then compile or update the sample frame and, if necessary, randomly select households to replace those that were absent. This period also enabled surveyors to familiarize themselves with local measurement units, converting them into standard metrics, inventory the crops produced in the village, and acclimate the villagers to the surveyors’ presence.

### Implementation phasing

The process was divided into three phases spanning ten months. The preparatory phase, lasting two to three months, ideally between April and July, involved the ROS coordination team refining the general and specific sections of the questionnaire. They also updated user manuals, technical documents for data collection, and budget projections. Supervisors assembled essential kits for field surveyors containing cooking tools, camping gear, and data collection materials. A unified ten-day training session was conducted for all supervisors to ensure consistency in methodologies, concepts, and data collection practices across all observatories.

The fieldwork phase required approximately one month in each observatory, at months adapted to the local context. It entailed filling out and verifying the questionnaires through interviews with the selected households. Prior to departing for their survey sites, the recruited surveyors, approximately ten for each observatory, underwent a week-long training session. Supervisors, who were ultimately accountable for the accuracy of the completed questionnaires, accompanied them and performed first consistency tests on the data collected. This phase was frequently divided, with observatories addressed in groups, considering variations in agricultural schedules, as well as constraints related to human resources and logistics.

The data entry, validation, and analysis phase included actions implemented from June to January. Surveys were conducted in accordance with the agricultural calendar to minimize disruption to rural household livelihoods, and to visit villages during less labor-intensive seasons. Given the variation of the agricultural calendar across agro-climatic zones, surveys were conducted over an extended period. Prior to the first data collection, the production of the input mask, the program for splitting and merging data files and the consistency-testing program were completed. Data from the first observatories to be surveyed were entered before the onset of data collection in observatories with later agricultural schedules.

### Measurement harmonization

The selection of measurement units raised a methodological issue. Among the peasant population, the units used to measure surfaces, weights, and volumes are not standardized and vary from village to village. Requiring peasant farmers and fishermen to answer in standard units, i.e., kilograms, liters, or hectares, was the surest way of losing touch with reality. Hence, the ROS adopted “peasant measurements” which varied from one observatory to another depending on farming practices, and were named differently in regional languages. Output quantities are measured in units like *daba* or *vata* (oilcans), *zinga* (a kind of metal drinking vessel), *sobika* (baskets), or cartloads. Rice-growing acreage can be quantified in terms of the number of transplanters, seed quantities, *kipa* (the number of seedlings to be planted), or carts of rice bales harvested in the field. These local measurement units were identified in each village and converted into standard weights and measures. This conversion process had to be conducted separately for each village due to variations in the meanings of the same name across different locations (for example, the *cans* or *daba* hold different volumes of fluid), and the absence of standardization of “peasant measurements” between villages. After each complete interview, the survey supervisor reviewed the questionnaire and converted the gathered “peasant measurements” into standard weights and measures. This ensured the mitigation of measurement errors that are common in surveys on rural households.

### Ethics

The study was approved by the Madagascar National Statistical Office (INSTAT) and conducted in collaboration with INSTAT and the Ministry of Agriculture, ensuring compliance with national statistical regulations and public administration decisions. These institutions are the legitimate authorities responsible for authorizing, administering, and vetting large surveys in the country.

At the beginning of each interview, informed consent was obtained orally from each respondent. Respondent agreed for their data to be used for public policy and research purposes, provided that their anonymity is maintained. If consent was not granted, the interview did not proceed, and a replacement household was selected. No minors were interviewed. To ensure that respondents are fully aware of the purpose of the ROS, one of the key principles of the system is the systematic presentation of survey results as soon as they become available in the villages concerned. At the same time, this principle of dissemination to the surveyed population ensures that they benefit from the ROS and have the information they need to address difficulties or make their voices heard.

Several measures were implemented to protect participant data. Direct identifiers were removed and replaced with sequential aliases, pseudonymizing the data. Indirect identifiers (e.g., village name, age, gender, professional activity) remain in the dataset, theoretically allowing re-identification by someone with prior knowledge of the households. However, given the low penetration of information technology and administrative systems in these rural areas, the risk of re-identification is minimal.

To strengthen confidentiality, the data is available under a Data Usage Agreement (DUA) that requires users to adhere to strict confidentiality rules, prohibits attempts to re-identify participants, and mandates the data destruction after the project concludes. Access to the microdata requires a confidentiality declaration, ensuring that only authorized researchers with legitimate purposes can access the data. The pseudonymization of the data, as well as its deposit in the DataSuds repository, is covered by a formal declaration (identifier #202) in the IRD’s registry of personal data processing, in compliance with the General Data Protection Regulation (GDPR), which governs IRD as the responsible data controller.

## Data Records

The data collection consists of two datasets and a code repository^19^. The rationale behind having two distinct datasets is to ensure that the ROS metadata and documentation are openly accessible, while also addressing the confidentiality concerns related to the raw data from household surveys. All direct identifiers have been removed from the raw data, yet it contains detailed information about household composition, incomes, expenses, and assets as well as individuals’ age, gender, education and activities. This level of detail poses a risk of re-identifying specific individuals, especially in small rural areas. To mitigate this risk and protect the confidentiality of sensitive information, the raw data from household surveys is stored separately in its own dataset.

The two datasets are hosted in the DataSuds platform. It is an instance of the Dataverse open-source research data repository software. It was created and is administered by the French Research Institute for Sustainable Development (IRD) to provide research partners in developing countries with open science infrastructure.

The source code is maintained in a GitHub repository. Authors can make further improvements and complements to this source code. Each version of the source code is versioned, allowing users to retrieve the state of the source code at the time of the validation, included in Table 3.

**Table 3:** Outline of the data records’ content (Source: authors).

Name and location	Content description	Format and details
Metadata and documentation dataset	- Data catalog	- xlsx and tab
	- Documentation catalog	- txt
	- ROS methodological material: surveyor manuals, questionnaires, and other technical documents	- pdf
	- Full list of publications produced from the ROS data	- bibtex and pdf
	- Publications of the ROS project that are not accessible otherwise	- pdf
Raw household survey dataset	- Data catalog	- xlsx and tab
	- One folder per year with, in each one: o Household composition, housing conditions, asset possession, land tenure, nutrition, food security, incomes and expenses. o Individual characteristics of household members: gender, age, education o Household member activities (with an in-depth analysis of farming practices), and finances o Additional annual or regional data collected in specific modules (e.g., fishing, vanilla production, natural disasters, hygiene, perinatal practices…)	- All raw data provided in two versions: o Original proprietary format (Stata) o Open format (tsv)
Source code repository	- Workflow to catalog the data and assess its quality, enhance its metadata and format - Data pseudonymization workflow - Workflow to geolocate raw data at village level based on toponyms	Quarto markdown notebook with R code sections for data processing

Formats are abbreviated in the table as follows: tabulation separated values (tsv), Excel Open XML Spreadsheet (xlsx) and portable document format (pdf).

## Technical Validation

This section delves into the processes and methodologies employed in the ROS to ensure high data quality standards. Additionally, we provide a retrospective assessment of panel attrition, an essential aspect for longitudinal studies.

### Data collection process

From the inception of the data collection in 1995, measures were implemented at every stage of the survey process to ensure the reliability of the data collected. The ROS was created during a period characterized by the multiplication of observatories in developing countries, often employing multidisciplinary approaches to implement thematic monographies^20^. However, these initiatives often lacked reliable longitudinal data suitable for quantitative analysis. In contrast, the observatories in Madagascar were established from the outset with a clear emphasis on economic and statistical expertise, making the production of consistent data series the main priority. While researchers from various disciplines, such as geography, economics, and anthropology, contributed to the ROS, the project has always emphasized the best practices in survey management, in particular concerning sampling, quality control, and data analysis.

Quality control during survey data collection was paramount. Each surveyor was limited to three daily household interviews, preventing rushing and ensuring comprehensive data collection. These interviews, typically involving the householder and their partner, lasted approximately two hours. At the end of each day, surveyors returned completed questionnaires to their supervisor, who assigned new interviews and conducted daily operations, including work schedule organization and questionnaire validation. Supervisors actively addressed errors or unclear responses and conducted consistency checks between questionnaire items. This close monitoring, facilitated by concentrating interviews in a limited area, was unique and crucial for maintaining data quality in challenging rural conditions. The continuous presence of surveyors in the villages throughout the survey periods, coupled with the active involvement of supervisors in all stages of fieldwork, from organization to data processing, characterized this network. This feature sets it apart from other rural surveys, where temporary surveyors and supervisors are often less integrated into the workflow. During the survey period, especially in the beginning and middle stages, a researcher supported the team to improve the quality of the data collection.

Additionally, validation tests were periodically conducted to ensure data accuracy. For instance, in 1996, a test compared the measured surface areas of fields with the acreage reported in interviews among a subset of households. This test tends to confirm the absence of consistent bias in interviewees’ statements, indicating a balance in any inaccuracies.

### Attrition

Significant levels of attrition are reported from the ROS data^21,22^. It is well known that such attrition levels are common in any longstanding panel survey^23^. Attrition has been extensively studied and understood by survey experts. There is a strong consensus that despite this phenomenon, panel data is useful and necessary for understanding population living standard dynamics^24^. The attrition rate is the percentage of households interviewed in the previous year that were not re-interviewed in the year of interest.

**Fig. 3:**
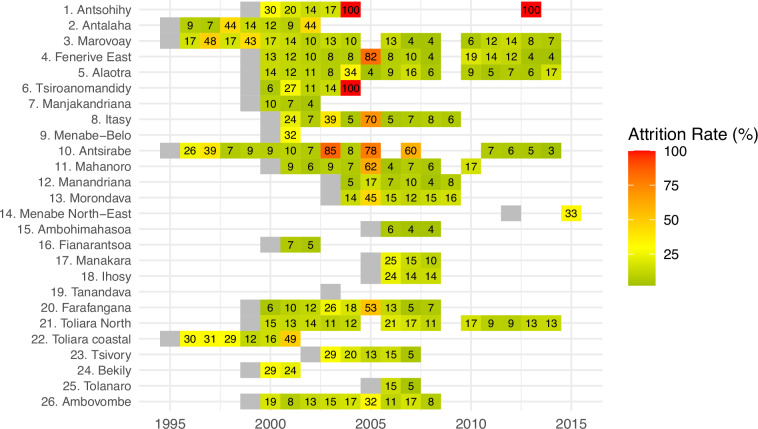
Attrition rates in ROS panels by observatory and survey round (Source: ROS raw data and author calculations). This heatmap displays attrition rates for each observatory across survey years from 1995 to 2015. The vertical axis lists the observatories, while the horizontal axis represents the years. Each square at the intersection of an observatory and a survey year denotes the attrition rate, with the color gradient varying from green (0% attrition) to red (100% attrition). The attrition rate for each year is rounded and displayed within the corresponding square. The initial survey year for each observatory is marked in gray, indicating the absence of prior data for attrition calculation.

Figure 3 depicts the attrition rate per year and per observatory. The six instances of annual attrition rates above 75% were likely induced by reshuffles of household identification codes or shifts in observatory sites. We are working to reconcile old and new identifications, and future dataset updates will include corresponding codes where possible. Excluding these outliers, the average annual attrition rate stands at 15%, aligning with developing countries’ standards and resulting in a cumulative attrition rate of 80% over ten years. In 2005, an IRD team conducted a tracking survey in one village of the Marovoay observatory (#3 in Fig. 1), successfully locating 59% of the attrited households^22^. Among these, 35% of households had split, and 65% had migrated out of the village. Other factors contributing to attrition, albeit rarely observed by ROS members, include the absence of the household during surveyor visits or the refusal to answer the survey. Nonetheless, it is important to note that attrition can introduce bias, requiring careful consideration in the analysis.

## Usage Notes

Access to the survey documentation and metadata is open, allowing any interested researcher to explore the dataset content, production process and associated materials. However, access to the raw survey data requires researchers to register, specify the purpose of data access, and commit to upholding the confidentiality rules outlined in the associated end-user license.

Given the number of variables and survey rounds, we recommend users to utilize the data catalog provided as a spreadsheet in the documentation and the raw data datasets. The data catalog contains the file name, table title, variable name, variable label and the years in which this variable is present in the dataset. The presence of labels facilitates the findings of relevant information using full-text search.

The GitHub repository associated to this paper serves as a technical appendix. It provides support materials in two formats: raw computational notebooks in markdown with R code chunks, and a web output. The web output is a HTML report featuring formatted text, embedded source code (indented and highlighted for readability), results of data analysis, and associated visualizations. This setup aims to be technically reproducible and didactically user-friendly for researchers exploring this data. The repository contains instructions to replicate all figures in the paper and includes a tutorial on georeferencing the data, useful for various types of analysis.

Users are advised to submit any questions or comments about the source code or data directly on the GitHub repository’s issues page (https://github.com/BETSAKA/Rural_obs_Madagascar/issues), instead of contacting the paper’s corresponding author via email. This approach promotes transparency as all issues and responses are publicly accessible and can be referenced by others facing similar challenges. It also helps build a collaborative knowledge base that benefits the user community.

## Data Availability

The data pseudonymization, data validation, and guidance for data georeferencing and analysis are available at https://github.com/BETSAKA/Rural_obs_Madagascar, under the MIT open-source license. This work used *R* 4.3.1 for the general data processing environment^25^, alongside the following packages: *tidyverse* 2.0.0 for data preparation and visualization^26^, *haven* 2.5.3 for reading and converting files from Stata format^27^, *labelled* 2.12.0 for generating the data catalog^28^, *sf* 1.0.14 for spatial data georeferencing^29^, *stringdist* 0.9.10 for toponym matching and data pseudonymization^30^, and *tmap* 3.3.4 for spatial data visualization^31^.
